# Clinical characteristics and registry-validated extended pedigrees of germline *TP53* mutation carriers in Denmark

**DOI:** 10.1371/journal.pone.0190050

**Published:** 2018-01-11

**Authors:** Ulrik Stoltze, Anne-Bine Skytte, Henriette Roed, Henrik Hasle, Bent Ejlertsen, Thomas van Overeem Hansen, Kjeld Schmiegelow, Anne-Marie Gerdes, Karin Wadt

**Affiliations:** 1 Clinical Genetics Dept., Rigshospitalet, Copenhagen, Denmark; 2 Pediatric Oncology Laboratory, Rigshospitalet, Copenhagen, Denmark; 3 Clinical Genetics Dept., Aarhus University Hospital, Aarhus, Denmark; 4 Clinical Genetics Dept., Odense University Hospital, Odensen, Denmark; 5 Pediatrics and Adolescent Medicine, Aarhus University Hospital, Aarhus, Denmark; 6 Oncology Clinic, Finsen Center Rigshospitalet, Copenhagen, Denmark; 7 Center of Genomic Medicine, Rigshospitalet, Copenhagen, Denmark; University of Texas MD Anderson Cancer Center, UNITED STATES

## Abstract

**Introduction:**

*TP53* mutation carrier (Li-Fraumeni Syndrome, LFS) cohort studies often suffer from lack of extensive pedigree exploration.

**Methods:**

We performed a nation-wide exploration of *TP53* mutation carrier families identified through all clinical genetics departments in Denmark. Pedigrees were expanded and verified using unique national person identification, cancer, cause of death, pathology, and church registries.

**Results:**

We identified 30 confirmed, six obligate and 14 assumed carriers in 15 families harboring 14 different mutations, including five novel and three *de novo* germline mutations. All but two (96%) developed cancer by age 54 years [mean debut age; 29.1 y., median 33.0 y., n = 26 (17F, 9M), range 1–54 y]]. Cancer was the primary cause of all deaths [average age at death; 34.5 years]. Two tumors were identified through registry data alone. Two independent families harbored novel c.80delC mutations shown to be related through an ancestor born in 1907. This exhaustive national collection yielded markedly fewer *TP53* mutation carriers than the 300–1,100 expected based on estimated background population frequencies.

**Conclusion:**

Germline *TP53* mutations in Denmark are likely to be drastically underdiagnosed despite their severe phenotype. Following recent advances in surveillance options of LFS patients, lack of pre-symptomatic testing may lead to the mismanagement of some individuals.

## Introduction

Li-Fraumeni Syndrome (LFS; Online Mendelian Inheritance in Man entry [OMIM] 151623) is an autosomal dominant cancer predisposition syndrome caused by germline mutations in *TP53* [1]. The known tumor spectrum of LFS comprises nearly all tissues, hazard ratios being especially increased for osteosarcomas, adrenocortical carcinomas (ACC), central nervous system (CNS) tumors, and soft tissue sarcomas, as well as breast cancer in younger women [2]. In four of the rare malignancies: childhood ACC, anaplastic rhabdomyosarcoma, choroid plexus carcinoma, and low hypodiploid acute lymphoblastic leukemia, *TP53* mutation carriers account for 40–80% of all patients [3–5].

LFS is suspected based on phenotype criteria and confirmed by identifying a pathogenic germline variant in *TP53*, however, germline mutations may be missing in some families with characteristic LFS patterns of cancer, and conversely as many as 20% of patients have *de novo* mutations and are thus without a LFS pattern family history of cancer [6].

The lifetime risk of cancer has been estimated at around 70% for men and approaching 100% for women, with approximately one third developing cancer before age 18 years [2,7]. LFS is believed to be markedly underdiagnosed. A recent germline genomic study of >1,000 childhood cancer patients identified 50 *TP53* mutation carriers, most of whom lacked a family cancer history supporting LFS, however the study was markedly enriched with patients with tumors within the LFS spectrum [5].

The International Agency for Research on Cancer (IARC) curates the most comprehensive database of germline *TP53* mutations with 1,827 confirmed carriers as of April 2016 (Version R18, http://p53.iarc.fr/). This relatively limited number of entries in the IARC database contrasts the anticipated frequency of *TP53* mutation carriers from 1 in 5,000 to 20,000 individuals in the general population [8,9]. This most likely stems from an underreporting of TP53 germline mutations from the diagnostic genetic laboratories, but also hints at a possible under-diagnosis of LFS globally.

Pathogenic mutations of the *TP53* gene can result in either loss-of-function or a gain-of-function/dominant negative effect. Dominant negative mutations in the DNA-binding domain blocks the function of the wild-type allele and are thus generally associated with cancer debut at an earlier age than loss-of-function mutations [2].

Sequencing of *TP53* is inconsistently offered to patients or families harboring LFS phenotypes. However, this tendency is shifting due to decreasing costs of sequencing, potential benefits of tumor surveillance, need for individualized chemo- and radiotherapy in patients with LFS, and emerging reproductive options including preimplantation genetic diagnosis [10–13].

## Methods

Information on *TP53* mutation carriers was obtained from all departments of clinical genetics in Denmark. Only families with clinically confirmed *TP53* mutations were included. In addition, one Danish family (family #11) was identified from the IARC database [14]. Access to electronic medical records (EMRs) was obtained by individual contact to the clinical genetics departments. EMRs included pedigrees taken and expanded at the time of LFS diagnosis, histological pathology reports of tested individuals with a cancer diagnosis, and laboratory reports of *TP53* sequencing. All pedigrees were expanded further through the unique identification numbers in The Danish Personal Identification Number Registry (CPR, every Danish citizen alive in 1968 or later) and/or Church registries. CPR-numbers are linked to first-degree relatives which allow for the extension of pedigrees, which was done by at least one generation above the oldest known affected individual wherever possible. Subsequently CPR numbers were linked to registry data from 1) the Danish Cancer registry (all cancer diagnoses from 1943 to 2014), 2) the Danish Cause of Death Registry (deceased after 1970 and before 2014), and 3) the Danish Pathology Registry (tissues described 1970 or later). Information on receptor status in breast cancers was included when available. CPR provided death status for subjects after 2014. Through church registries and censuses, two families not previously known to be related (#5 and #13) were backtracked to a shared female relative born in 1907.

Starting in June 2012, a gene panel has been used in the oncological management of breast cancer (BC) patients at one Danish hospital to screen women for mutations in genes associated with increased risk of breast cancer, including *BRCA1*, *BRCA2*, *CDH1*, *PTEN*, *RAD51C* and *TP53*. The criteria for panel testing were <40 years at BC diagnosis, triple negative BC, bilateral or multifocal BC, or BC and family history of breast/ovarian cancer. One individual (subject II.2 in family #7) was 41 years at diagnosis and had no first- or second-degree family members with cancer, however, more distant relatives had cases of middle-age breast cancer, and it was decided to test the youngest individual in the family. LFS families identified through BC panel testing were compared to families diagnosed through other investigations, to examine pheno- and genotype differences.

Putative dominant negativity was defined as a pathogenic missense mutation inside the DNA-binding domain (DBD) of the *TP53* gene, and cancer debut age in individuals carrying *TP53* missense mutations inside the DBD vs. individuals carrying *TP53* mutations outside or non-missense *TP53* mutations inside the DBD, was tested (S1 Fig).

TP53 analysis was performed by Sanger sequencing until 2012 and from 2012 and onwards by next-generation sequencing (NGS) analysis as recently described [15]. Briefly, the NGS sequence capture library (Roche NimbleGen) was designed to capture all exons from the *TP53* gene (NM_000546) as well as five other genes (*BRCA1*, *BRCA2*, *CDH1*, *PTEN*, *RAD51C*). Sequencing was performed on a MiSeq (llumina) to an average coverage of at least 100x. Sequencing data were analyzed using Sequence Pilot software (JSI medical systems). Variants were called if the allele frequency was above 25%, except for formalin-fixed paraffin-embedded (FFPE) tissue where specific mutations were examined in all sequence reads. Copy number variations were detected by multiplex ligation-dependent probe amplification (MRC-Holland) analysis or by analysis of NGS data. All nucleotide variants were verified by Sanger sequencing on an ABI 3730 DNA Analyzer using DNA purified from a new blood sample.

Untested LFS relatives were classified as either 1) obligate carriers or 2) probable carriers, defined as untested relatives of a carrier with either pathogenomic cancers (e.g. ACC) or onset of a cancer on the LFS spectrum before age 46 or multiple primaries consistent with LFS at any age.

The project is approved by the Danish Health Authority and the need for individual consent was waived. Based on the waiver the ethical committees in Denmark determined that the study was not subject to their approval (jr.n. 15012065).

## Results

**Fig 1:**
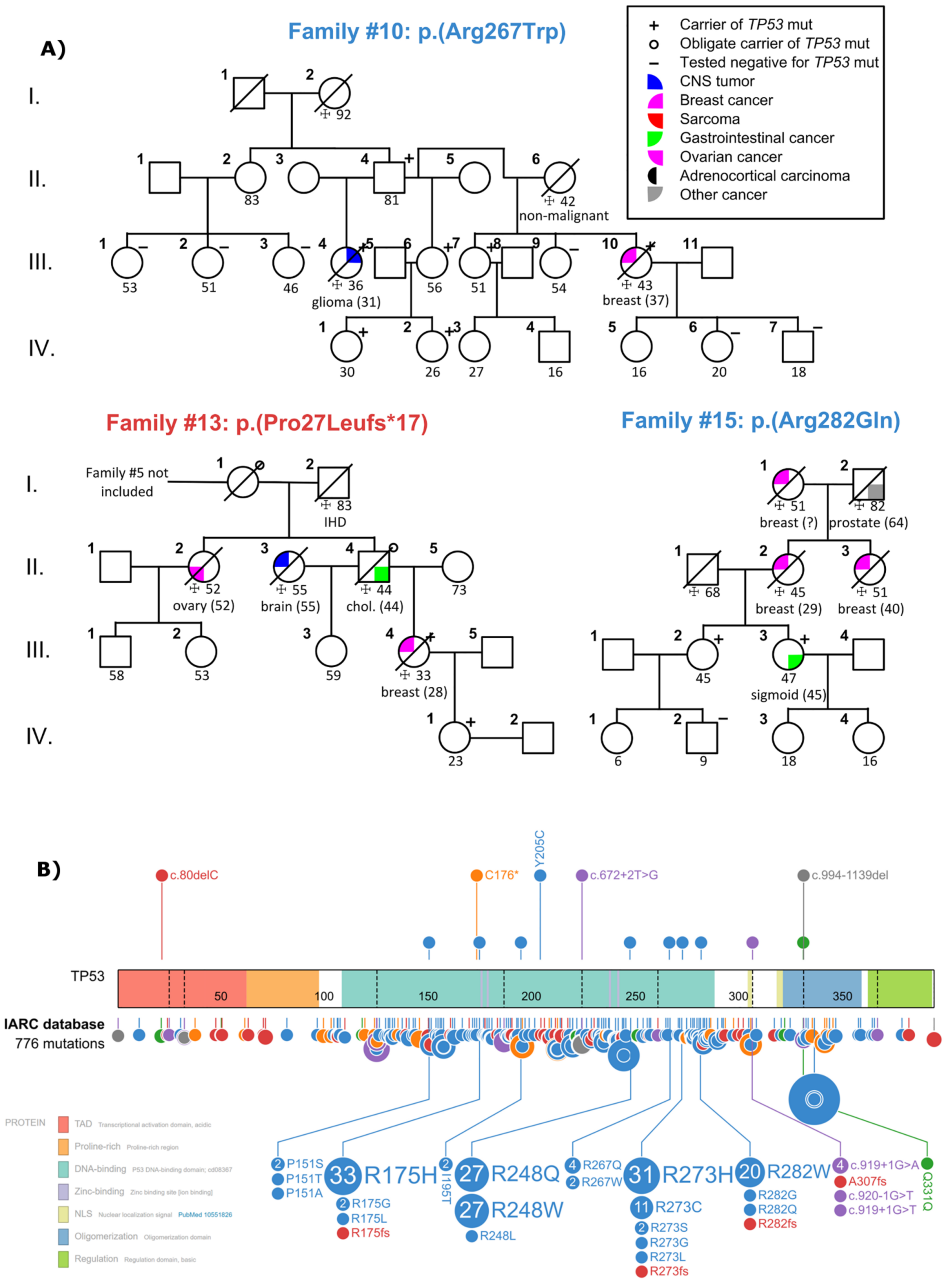
(A) Pedigrees of three Danish families harboring germline *TP53* mutations. (B) NM_000546 isoform of the *TP53* gene protein product showing the mutations found in this study (upper track) and all published mutations from the IARC database (lower track). In the upper track, 5 novel germline mutations described in this study are expanded. In the lower track, mutations from 9 loci in the IARC database are expanded, corresponding to the 9 non-novel mutation sites described in this study. 14 IARC mutations were left out as they were either complex (13) or not classified (1). Variant colors: blue, missense, orange, nonsense, purple, splice region, red, frameshift, green, silent, grey, protein deletion.

The clinical genetics departments provided a total of 15 LFS families harboring 14 different mutations; five novel germline mutations not previously described in the IARC database, two mutations seen in single families (one and two confirmed cases each), three mutations seen in two families, and four frequently reported mutations (Fig 1 and Table 1) [16]. One of the novel mutations (c.80delC) was identified in two independently diagnosed families (#5 and #13, see Methods) that were subsequently shown to be distantly related. Time since genetic diagnosis in the families ranged from 2002 to 2016, with all but four families being diagnosed during or after 2012.

**Table 1:** IARC database characteristics of germline *TP53* mutations observed in both IARC and in this study (families with more than 10 confirmed carriers in the IARC database are in bold).

Fam #	Mutation	# of families	# of carriers	Perc. with cancer	Mean age at first cancer	Perc. with >1 cancer	Perc. with cancer <18y	Perc. by cancer site: breast/brain/ST/bone/other
**2**	**c.524G>A, p.(Arg175His), exon 5, missense**	**33**	**54**	**88.9% (48)**	**21.9y**	**38.9% (21)**	**29.6% (16)**	**28%/17%/16%/9%/29% (75)**
4	c.993G>A, p.?, exon 9, splice	1	2	100% (2)	21.5y	0.0% (0)	50.0% (1)	50%/0%/0%/0%/50% (2)
**6**	**c.818G>A, p.(Arg273His), exon 8, missense**	**31**	**42**	**90.5% (38)**	**20.5y**	**40.5% (17)**	**35.7% (15)**	**22%/10%/24%/16%/29% (63)**
8	c.584T>C, p.(Ile195Thr), exon 6, missense	2	2	100% (2)	22.5y	50% (1)	0.0% (0)	33%/33%/0%/0%/33% (3)
**9**	**c.742C>T, p.(Arg248Trp), exon 7, missense**	**27**	**58**	**93.1% (54)**	**19.8y**	**39.7% (23)**	**39.7% (23)**	**28%/25%/7%/7%/33% (88)**
10	c.799C>T, p.(Arg267Trp), exon 8, missense	2	2	50.0% (1)	N/A	0.0% (0)	0.0% (0)	100%/0%/0%/0%/0% (1)
11	c.919+1G>A, p.?, intron 8, splice ^ᵻ^	4	7	71.4% (5)	14.8y	28.6% (2)	28.6% (2)	28%/14%/28%/14%/14% (7)
14	c.451C>T, p.(Pro151Ser), exon 5, missense	2	2	100% (2)	12.5y	50% (1)	50% (1)	25%/0%/0%/0%/75% (4)
15	c.845G>A, p.(Arg282Gln), exon 8, missense	1	1	100% (1)	1y	100% (1)	100% (1)	0%/0%/0%/0%/100% (2)

Table was constructed using data from The International Agency for Research on Cancer (IARC) *TP53* mutation registry version R18, April 2016. Families #1, #3, #5, #7, #12 and #13 are not listed as they carried germline *TP53* mutations not described in the IARC database. Mutations are described according to NM_000546.5. N is given in parenthesis for each percentage.

^**ᵻ**^ This mutation includes data also presented in this study. Perc., percentage, #, number, <18y, under 18 years of age, ST, soft tissue.

The 15 families contained 30 confirmed, six obligate, and 14 probable carriers, collectively diagnosed with 50 malignant tumors (excluding four non-melanoma skin cancers). 33 (66%) cancers were observed among 26 confirmed or obligate *TP53* carriers and another 17 (34%) observed in 14 probable carriers (Table 2 and S1 Table).

**Table 2:** Clinical and mutational data of families with 36 confirmed or *obligate* germline *TP53* mutations carriers.

Fam #	Pt #	Sex	Tumor(s)^ᵻ^	Age at Dx	2^nd^	Age at death	Criteria	Mutation	Domain	IARC Fq ^ᵻ ᵻ^	Note
1	III.2	F	Anaplastic astrocytoma (H)	18	-	-	LFS	c.614A>G, p.(Tyr205Cys), exon 6, missense	DNA-binding	novel	
	III.4	F	Liposarcoma (H)	7	-	-					
	*II*.*4*	*F*	*IDC*, *ER+*, *HER- (H)*	*35*	-	*37*					
2	V.1	M	Sarcoma of thorax (R)	8	-	9	LFS	c.524G>A, p.(Arg175His), exon 5, missense	DNA-binding	54(33)	
	IV.1	M	Adrenocortical carc. (H)	28	-	31					
3	III.5	M	Leiomyosarcoma (H) Pancreatic (H)	38	8	47	LFS	c.672+2T>G, p.?, intron 6, splice	-	novel	
	III.1	F	IDC, ER-, HER+ (H)	21	-	-					
	*II*.*2*	*M*	*Melanoma (H)* *Leiomyosarcoma (H)*	*46*	*12*	*60*					
4	III.2	F	IDC, ER+ (H) CRC acendens (H)	35	6	43	Chompret	c.993G>A, p.?, exon 9, splice	Oligomeriz.	2(1)	
5	IV.2	F	IDC, ER+, HER+ (H)	23	-	-	LFS	c.80del, p.(Pro27Leufs*17), exon 4, frameshift	several	novel	
	*III*.*2*	*F*	*IDC*, *ER- (H)* *Ovarian seropap*. *carc*. *(D)*	*35*	*16*	*54*					
	*II*.*2*	*M*	*Adenocarc*. *oesoph*. *(H)*	*54*	-	*54*					
6	III.2	F	IDC, ER+, HER+ (H)	53	-	-	none	c.818G>A, p.(Arg273His), exon 8, missense	DNA-binding	42(31)	(putativemosaic)
7	II.2	F	IDC, ER+, HER+ (H)	41	-	-	none	c.528C>A, p.(Cys176*), exon 5, nonsense	several	novel	(de novo)
8	III.2	F	IDC, ER+ (H)	38	-	56	LFS	c.584T>C, p.(Ile195Thr), exon 6, missense	DNA-binding	2(2)	
9	II.1	F	Choroid plexus carc. (H)	12	-	-	Chompret	c.742C>T, p.(Arg248Trp), exon 7, missense	DNA-binding	58(27)	(de novo)
10	III.10	F	IDC. ER+, HER+ (H)	37	-	43	Chompret	c.799C>T, p.(Arg267Trp), exon 8, missense	DNA-binding	2(2)	
	III.4	F	Oligodendroglioma II (H)	31	-	36					
11	III.2	M	Leydig cell tumor (H) PNET (H)	1	5	7	Chompret	c.919+1G>A, p.?, intron 8, splice	-	7(4)	[15]
	II.2	M	Leiomyosarcoma (R)	45	-	-					
12	III.2	F	Anaplastic RMS (H) Glioblastoma (H)	2	11	-	LFS	c.994_1139del, p.(Ile332*), exon 9, nonsense	several	novel	
	II.2	F	Glioblastoma (H)	28	-	29					
13	III.4	F	IDC, ER+ (R)	28	-	33	Eeles	Shown to have the same mutation as Fam #5			
	*II*.*4*	*M*	*Ductus choled*. *carc*. *(R)*	*44*	-	*44*					
14	II.2	M	Fibrous histiocytoma (H) Dermafibrosarcoma (H)	3	2	17	None	c.451C>T, p.(Pro151Ser), exon 5, missense	DNA-binding	2(2)	
15	III.3	F	CRC, sigmoid (H)	45	-	-	Eeles	c.845G>A, p.(Arg282Gln), exon 8, missense	DNA-binding	1(1)	

Mutations are described according to NM_000546.5. Nine unaffected *TP53* mutation carriers (one male) from 5 different families and one obligate carrier with unknown cause of death and cancer status are not included in the table. The nine unaffected carriers had an average age at time of study of 41,6 years [range 17-81y].

^**ᵻ**^ strongest evidence for disease given in parentheses with H, histology, R, registry, D, death certificate,

^**ᵻ ᵻ**^ Fq, frequency; number of times mutation is seen in IARC *TP53*-database with number of confirmed carriers (number of families in parentheses) novel indicates novelty on a germline level. Cancer was the primary cause of all deaths. *Italics* indicate obligate carrier. 2^nd^, years until second primary cancer following cancer debut,

*, nonsense codon, del, deletion, PNET, primitive neuro-ectodermal tumor, CRC, colorectal cancer, RMS, rhabdomyosarcoma, IDC, invasive ductal carcinoma, ER, estrogen receptor positive, HER, herceptin receptor positive, +/-, positive/negative (where receptor status is not given it is unknown), NOS, not otherwise specified, carc., carcinoma, adenocarc., adenocarcinoma, oesoph., oesophageal, seropap., seropapillary, choled., choledochus.

Age at diagnosis of first cancer among the 26 affected carriers, including obligate carriers, was a mean of 29.1 years and median of 33.0 years, n = 26 (17F, 9M), [range 1–54 years] and all fatalities occurred with cancer being the primary cause of death (average; 34.5 years [7–60], n = 16). We found no age difference in cancer debut between carriers with and without putative dominant-negative *TP53* mutations (25.9 versus 28.7 years, p = 0.65) (S1 Fig). Anticipation in LFS families has been previously demonstrated, however, we were unable to show any significant anticipation, likely due to the size of the cohort [17] (S2 Table).

A recent study established a link between development of choriocarcinoma in non-carrier mothers pregnant with a *TP53* mutation carrier fetus [13]. Among the *TP53* mutation-negative females in this study who 1) had a *TP53* mutation-positive partner and 2) gave birth to offspring with either confirmed or unknown *TP53* mutation status, no cases of choriocarcinoma were found (N = 10).

Among gene panel screening of 982 female BC (breast cancer) patients [average age; 48.2 years], two were found to harbor a *TP53* mutation, indicating a frequency of germline TP53 mutations in 0.2% of women with BC fulfilling the criteria for genetic screening. In total eight families were identified solely due to their family BC history (families #4, #5, #6, #7, #8, #10, #13, and #15). These families had a significantly older average age of first cancer when compared to the seven LFS families identified through other means (35.8 vs. 21.7 years, p = 0.01).

## Five novel germline *TP53* pathogenic variants

Family #1 harbored a c.614A>G, p.(Tyr205Cys), missense mutation in exon 6. This locus is a hotspot for somatic mutations in the *TP53* gene with 86 reported somatic mutations, including in five astrocytomas. Transcriptional assays of the somatic variant have classified it as non-functional [18]. The germline variant was found in all three daughters of the mother (subject II.4). The mother developed invasive ductal carcinoma (IDC) of the breast at age 35. Two of her daughters developed liposarcoma and astrocytoma at ages 7 and 18 years, respectively.

Family #3 harbored a germline splice donor site mutation. The c.672+2T>G, p.?, intron 6, splice mutation was found in subject III.5, who developed a leiomyosarcoma and pancreatic cancer at age 38 and 46 years, respectively. His father developed both melanoma and leiomyosarcoma at age 46 and 58 respectively, and his sister had a melanoma at age 46 (not included in the Table 2 and S1 Table as she did not fulfill the criteria for being a probable carrier), and a daughter with IDC at age 21.

Family #7 harbored a c.528C>A, p.(Cys176*), nonsense mutation in exon 5 leading to a stop-gain at codon 176. The mutation was found in a woman (II.2) with ER- and HER-positive IDC of the breast at age 41. Her parents (I.1 and I.2) were both tested negative for the mutation, suggesting a *de novo* mutation although germline mosaicism cannot be excluded.

Family #12 harbored a c.994_1139del, p.(Ile332*), nonsense mutation in exon 9, detected in a girl (III.2) who developed anaplastic rhabdomyosarcoma at age 2 and a second primary (astrocytoma) at age 13. Her mother (II.2) also carried the deletion and was diagnosed with glioblastoma at age 28 years. The mutation was likely inherited from her mother (I.2), diagnosed with bilateral breast cancer at 23.

Family #5 and #13 both harbored a c.80del, p.(Pro27Leufs*17), frameshift mutation in exon 4 leading to a premature stop at codon 43. The mutation was first found in a woman (IV.2) with ER- and HER-positive IDC of the breast at age 23, who had a brother (IV.5) diagnosed with rhabdomyosarcoma at age one year. Their mother (III.2) had IDC of the breast and ovarian papillary serous cystadenocarcinoma at age 35 and 51 respectively. Subject III.2 had a sister (III.3) who developed ovarian papillary serous cystadenocarcinoma, and her daughter (IV.2) was diagnosed with IDC of the breast at age 22 years. The dual observation of this novel mutation initiated the linkage of these families to a common female ancestor, established through Danish church registries. She was born in 1907 and likely died sometime before 1970, meaning she reached a maximum age of 63. Her cause of death and cancer status is unknown.

The five variants described above have, to our knowledge not been reported before at the germline level, but four of them (c.614A>G, c.80delC, c.528C>A, c.672+2T>G) have been identified as somatic mutations in various cancers.

Of the seven non-Danish Scandinavian mutations registered in the IARC LFS database only the c.993G>A mutation, resulting in a silent p.Q331Q amino acid change, overlapped with the mutations reported in this study. A previous report predicted this mutation to cause a loss of the splice donor signal and likely skipping of exon 9. Expression arrays indicated that the mutation results in an unstable mRNA transcript degraded by nonsense-mediated decay. [19] The mutation’s pathogenicity is further evidenced by family #4 in this study, that fulfills the Chompret criteria. No relationship between the families could be established.

By using registries to validate or discover cancers, 10 unreported tumors were discovered. These included eight non-melanoma skin cancers/cervix dysplasia found among both *TP53* mutation carriers and non-carriers, and excluded from our data due to their high rate of sporadic occurrence in the general population. The remaining two occurred in *TP53* mutation carriers and included a sarcoma of the thorax in an eight-year-old boy (V.1, family #2), and a leiomyosarcoma in a 45-year-old male (II.2, family #11).

## Discussion

This study found 50 confirmed, obligate, or probable *TP53* mutation carriers, yet the reported *TP53* mutation general population incidences of 1 in 5,000–20,000 should yield between 285 and 1,141 carriers in Denmark (population: 5,7 million). This could indicate that *TP53* mutations are underdiagnosed in Denmark, partly reflecting that *TP53* mutations are frequently found in cancer patients without significant family histories. If accurate this could be due to a higher frequency of de novo mutations and mosaicism, a lower penetrance compared to previous reports or, as shown for BRCA mutation carriers in previous studies, limited family structure defined as fewer than 2 first- or second-degree relatives surviving beyond 45 years in either lineage [5,20,21]. However, the relevance of the limited family structure differs for *TP53* mutation carriers as compared to BRCA mutation carriers, because *TP53* mutations have a markedly higher penetrance in males and carriers have a markedly lower age at first cancer diagnosis, typically debuting before 45 years of age. Furthermore, the testing of *TP53* mutation status has historically been limited in the clinically, even in cases where suspicion of LFS was raised. Testing was deferred due to limited possibilities of surveillance, and at the time no evidence of reduced mortality following genetic testing existed.

For *TP53* mutation carriers, the question of tumor surveillance is highly relevant. Among the 11 *TP53* mutation carriers in this study who, at the time of genetic investigation, hadn’t had cancer, two subsequently developed cancer, and could potentially have benefited from a cancer surveillance program. Recent results from use of the extensive “Toronto protocol”, which includes yearly whole-body and cerebral MRIs, indicated a survival benefit in *TP53* mutation carriers with 5-year overall survival rates of 89% in the surveillance group versus 60% in the non-surveillance group. However, the study was non-randomized and the two groups had remarkably and inexplicably different tumor incidences, (49% and 88% in surveillance versus non-surveillance groups, respectively). This is counterintuitive as one would expect similar or higher incidence in the surveillance group [11], however several of the lesions identified in the surveillance group were in fact pre-cancerous or low-grade tumors, with early detection leading to curative surgical treatment. Conclusively, the benefit of comprehensive surveillance programs awaits further data. One study has shown that, a targeted surveillance approach for ACC in *TP53* mutation carriers before age 15, decreases at-diagnosis tumor size with a trend towards lower stage and increased surgical cure in the surveillance group [22]. Numerous countries have adopted versions of comprehensive surveillance programs and the American Association for Cancer Research has recently issued a recommendation for screening of individuals with LFS [23]. Furthermore, it is widely believed that female *TP53* mutation carriers could benefit from breast cancer surveillance similarly to *BRCA1/2* mutation carriers, yet using non-irradiating surveillance such as MRI and ultrasound [24]. Of the 10 female TP53 mutation carriers with breast cancer we have estrogen receptor (ER) and Herceptin receptor (HER) status of 6 cancers. Four of six breast cancers were ER+ and HER+ whereas one was ER+, HER-, and one was ER-, HER+. This underscores previous reports that receptor status in early onset breast cancer should not necessarily dissuade panel testing [2,25].

Knowledge of *TP53* mutation carrier status has other implications for health management. As *TP53* mutations causes DNA instability it has been theorized that germline mutations in this gene increases risk of radiation-induced cancers. This theory is supported by observations in a cohort study where secondary cancers were observed at or near the site of radiation [2]. Furthermore, reproduction possibilities with either preimplantation diagnostics or prenatal diagnosis play an increasing role in fertile *TP53* mutation carriers [26]. Interestingly, knowledge of an underlying germline *TP53* mutation may also help guide diagnosis, as demonstrated in subject II.1 in family #9, who was shown to have a *de novo* R248W missense mutation. The child presented with a brain tumor of uncertain histological type and unknown origin, and the identification of a germline *TP53* mutation supported the diagnosis of a choroid plexus carcinoma.

While this study highlights the severe phenotype of LFS, we also observed a male mutation carrier (II.4, family #10) who, at the time of this study, had reached 81 years of age without developing cancer. Among his four daughters who have inherited the mutation, two developed cancer at 31 and 37 respectively, while two are 51 and 56 and unaffected. Collectively the family has 7 documented carriers, without a single occurrence of childhood cancer.

Our study had several shortcomings, including an ascertainment bias due to the incorporation of the breast cancer gene panel screening in the clinical setting. Furthermore, a few historical cases of LFS in Denmark may have been overlooked, however, these are likely to be small and without recent instances of cancer.

In conclusion, this detailed nation-wide curation of known LFS families identified 5 novel germline mutations, and emphasized the likelihood that such families are critically underdiagnosed. This report highlights the importance of raising awareness of LFS among health-care professionals to ensure that patients and physicians can make informed decisions about treatment, follow-up and reproduction. Following the recent advances in surveillance possibilities of LFS patients, the use of only strict clinical criteria and lack of pre-symptomatic testing may lead to the mismanagement of some individuals or families. Studies offering population-based genetic screening to patients with tumors within the LFS spectrum are needed, to further elucidate the prevalence and diagnostic yield as compared to the current approach based largely on strict clinical criteria.

## Supporting information

S1 Fig Plot of cumulative cancer debut age (in years) distribution in carriers of *TP53* mutations. The plot shows 8 missense mutations inside the DNA-binding domain of the *TP53* gene (p.(Pro151Ser), p.(Arg175His), p.(Ile195Thr), p.(Tyr205Cys), p.(Arg248Trp), p.(Arg267Trp), p.(Arg273His), p.(Arg282Gln)) vs. 6 mutations that are either outside of the DNA-binding domain or non-missense mutations (c.672+2T>G, p.?, c.993G>A, p.?, c.80del, p.(Pro27Leufs*17), c.528C>A, p.(Cys176*), c.919+1G>A, p.?, c.994_1139del, p.(Ile332*)).
(TIFF)

S1 Table Clinical and mutational data of 14 probable germline TP53 mutation carriers. Mutations are described according to NM_000546.5. ᵻ strongest evidence for disease given in parentheses with H, histology, R, Registry, D, Death certificate, F, family history. ᵻ ᵻ Number of times mutation is seen in IARC database with number of confirmed carriers (number of families in parentheses). Cancer was the primary cause of all deaths. All mutations are assumed to be inherited from or passed on to confirmed/obligate carriers in that family. 2nd, years until second primary cancer following debut, del, IDC, invasive ductal carcinoma, PNET, primitive neuro ectodermal tumor, NOS, not otherwise specified, bilat., bilateral, carc., carcinoma, dx., right-sided, sin., left-sided, ER, estrogen receptor, +/-, positive/negative (where receptor status is not given it is unknown).
(DOCX)

S2 Table Mean age in years at first cancer onset in different generations of LFS families with 1–4 documented generations. Upper case N indicates the number of generations of affected carriers available for study, lower case n indicates number of tumors used for each calculated mean age. Each row is the mean age of first malignant tumor onset for generations as given in the left column.
(DOCX)
