# Supplementary figures and images for: Clinical characteristics and registry-validated extended pedigrees of germline TP53 mutation carriers in Denmark
Source: PLoS One. 2018 Jan 11;13(1):e0190050. doi: 10.1371/journal.pone.0190050 (PMC5764253; doi:10.1371/journal.pone.0190050)

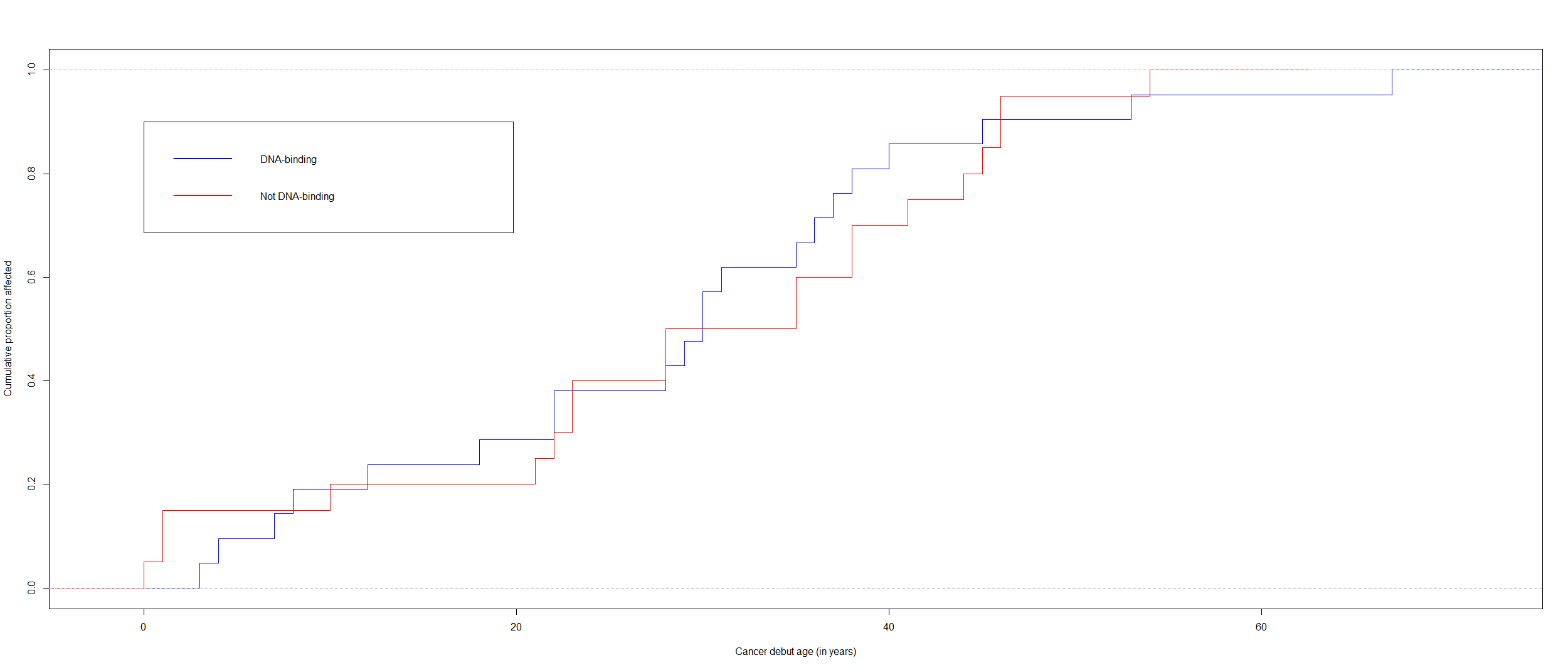

Supplement: S1 Fig — The plot shows 8 missense mutations inside the DNA-binding domain of the TP53 gene (p.(Pro151Ser), p.(Arg175His), p.(Ile195Thr), p.(Tyr205Cys), p.(Arg248Trp), p.(Arg267Trp), p.(Arg273His), p.(Arg282Gln)) vs. 6 mutations that are either outside of the DNA-binding domain or non-missense mutations (c.672+2T>G, p.?, c.993G>A, p.?, c.80del, p.(Pro27Leufs*17), c.528C>A, p.(Cys176*), c.919+1G>A, p.?, c.994_1139del, p.(Ile332*)). (TIFF) [file pone.0190050.s001.tiff]
