# Supplementary material for: Clinical characteristics and registry-validated extended pedigrees of germline TP53 mutation carriers in Denmark
Source: PLoS One. 2018 Jan 11;13(1):e0190050. doi: 10.1371/journal.pone.0190050 (PMC5764253; doi:10.1371/journal.pone.0190050)
Supplement: S1 Table — Mutations are described according to NM_000546.5. ᵻ strongest evidence for disease given in parentheses with H, histology, R, Registry, D, Death certificate, F, family history. ᵻ ᵻ Number of times mutation is seen in IARC database with number of confirmed carriers (number of families in parentheses). Cancer was the primary cause of all deaths. All mutations are assumed to be inherited from or passed on to confirmed/obligate carriers in that family. 2nd, years until second primary cancer following debut, del, IDC, invasive ductal carcinoma, PNET, primitive neuro ectodermal tumor, NOS, not otherwise specified, bilat., bilateral, carc., carcinoma, dx., right-sided, sin., left-sided, ER, estrogen receptor, +/-, positive/negative (where receptor status is not given it is unknown). (DOCX) [file pone.0190050.s002.docx]

| **Fam #** | **Pt #** | **Sex** | **Tumor(s) ^ᵻ^** | **Age at Dx** | **2^nd^** | **Age at death** | **Criteria** | **Mutation** | **Domain** | **Fq ^ᵻ ᵻ^** |
| --- | --- | --- | --- | --- | --- | --- | --- | --- | --- | --- |
| 2 | III.3 | M | Glioblastoma (H) | 28 | - | 28 | LFS | c.524G>A, p.(Arg175His), exon 5, missense | DNA-binding | 81(33) |
| 3 | I.2 | F | IDC, dx. (H)  Breast cancer, NOS, sin. (F) | 45 | 4 | 49 | LFS | c.672+2T>G, p.?, intron 6, splice | - | novel |
| 3 | IV.3 | F | Adrenocortical carc. (H) | 0 | - | - |  |  |  |  |
| 4 | II.2 | F | IDC, ER- (H) Truncal sarcoma (H)  PNET (H) | 67 | 3 | 70 | Chompret | c.993G>A, p.?, exon 9, splice | Oligomeriz. | 2(1) |
| 5 | III.3 | F | Ovarian carc. (R) | 38 | - | 40 | LFS | c.80del, p.(Pro27Leufs*17), exon 4, frameshift | several | novel |
|  | IV.5 | M | Rhabdomyosarcoma (R) | 1 | - | 3 |  |  |  |  |
|  | IV.7 | F | IDC, ER- (H) | 22 | - | 24 |  |  |  |  |
| 8 | I.2 | F | Breast cancer, NOS (F) | 30 | - | 31 | LFS | c.584T>C, p.(Ile195Thr), exon 6, missense | DNA-binding | 3(2) |
|  | II.2 | F | Breast cancer, NOS (F) | 30 | - | 34 |  |  |  |  |
|  | III.4 | M | Osteosarcoma (H) | 36 | - | 39 |  |  |  |  |
|  | IV.4 | F | Medullablastoma (H) | 4 | - | 5 |  |  |  |  |
| 12 | I.2 | F | Bilat. breast cancer, NOS (R) | 23 | - | 32 | LFS | c.994_1139del, p.(Ile332*), exon 9, nonsense | several | novel |
| 15 | II.2 | F | Breast cancer, NOS (F) | 29 | - | - | Eeles | c.845G>A, p.(Arg282Gln), exon 8, missense | DNA-binding | 1(1) |
|  | II.3 | F | Breast cancer, NOS (F) | 40 | - | - |  |  |  |  |
