# Supplementary material for: Clinical characteristics and registry-validated extended pedigrees of germline TP53 mutation carriers in Denmark
Source: PLoS One. 2018 Jan 11;13(1):e0190050. doi: 10.1371/journal.pone.0190050 (PMC5764253; doi:10.1371/journal.pone.0190050)
Supplement: S2 Table — Upper case N indicates the number of generations of affected carriers available for study, lower case n indicates number of tumors used for each calculated mean age. Each row is the mean age of first malignant tumor onset for generations as given in the left column. (DOCX) [file pone.0190050.s003.docx]

|  | | | | |
| --- | --- | --- | --- | --- |
| Generation | N=4, two families | N=3, Five families | N=2, Four families | N=1, 4 families |
| 1 | 37.5 (+/- 10.6), n=2 |  |  |  |
| 2 | 38 (+/- 11.3), n=2 | 37.8 (+/- 12.5), n=5 |  |  |
| 3 | 33.3 (+/- 8.2), n=4 | 32.3 (+/-5.3), n=7 | 49 (+/- 16.4), n=3 |  |
| 4 | 2 (+/- 2.8), n=1 | 21.6 (+/-14.7), n=5 | 21.5 (+/-15.2), n=6 | 27.3 (+/-23.6), n=4 |
